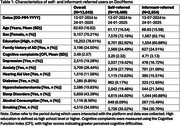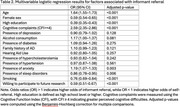# Subjective cognitive decline: Comparing users concerned about their own cognitive function vs. those referred by an informant

**DOI:** 10.1002/alz70857_106456

**Published:** 2025-12-25

**Authors:** Federica Cacciamani, Graziella Mangin Laignel, Audrey Gabelle, Nicolas Beaume, Mahinthan Kengatharan, Igor Koval, Stanley Durrleman

**Affiliations:** ^1^ Qairnel, Paris, France, France; ^2^ ARAMISLab, Sorbonne Université, Institut du Cerveau‐Paris Brain Institute‐ICM, CNRS, Inria, Inserm, AP‐HP, Hôpital de la Pitié Salpêtrière, Paris, France, France; ^3^ Université de Montpellier, Montpellier, France; ^4^ Memory Resource and Research Center of Montpellier, CHU de Montpellier, Hôpital Gui de Chauliac, Montpellier, France

## Abstract

**Background:**

Subjective cognitive decline (SCD) refers to a perceived worsening of cognitive abilities and is a common reason older adults seek medical advice. It can be reported by individuals themselves (self‐reported) or by someone close to them (informant‐reported), with the latter potentially indicating a higher risk of Alzheimer's disease and related disorders (ADRD), as suggested by recent research. DocMemo (docmemo.fr and docmemo.com) is a digital platform designed to support individuals with cognitive concerns and their informants by providing cognitive screening, educational resources, and research participation opportunities. This study aims to compare self‐ and informant‐referred individuals and examine how their referral type is associated with established dementia risk factors.

**Method:**

DocMemo users were classified as self‐referred or informant‐referred based on their responses to a cognitive complaint questionnaire. We performed multivariate logistic regression to assess the association between referral type (self vs. informant) and demographic, cognitive, psychological, health‐related, and lifestyle factors, adjusting for multiple comparisons.

**Result:**

A total of 13,043 users were analyzed (self‐referred: 10,489; informant‐referred: 2,554), with their characteristics detailed in Table 1.

Individuals with high cognitive complaints and older age were respectively 2.6 and 1.6 times more likely to be referred by an informant rather than self‐refer (all *p* <0.001). Anxiety was also associated with a higher likelihood of informant referral (OR=1.19, *p* = 0.003).

Conversely, females (OR=0.59, *p* <0.001), individuals with higher education (OR=0.39, *p* <0.001), those with sleep disorders (OR=0.86, *p* = 0.006), and smokers (OR=0.76, *p* <0.001) were less likely to be informant‐referred. Additionally, individuals willing to participate in further research were less likely to be informant‐referred (OR=0.59, *p* <0.001).

Other factors, including depression, alcohol consumption, diabetes, family history of AD, hearing aid use, hypercholesterolemia, and hypertension, were not significantly associated with referral type (*p* >0.05). Model results are reported in Table 2.

**Conclusion:**

Informant‐referred users exhibited higher AD risk profiles, aligning with established modifiable risk factors for dementia. Their profile suggests a population with substantial vulnerability, reinforcing the value of informant reports in identifying high‐risk individuals for early intervention. Recognizing this is crucial for optimizing screening strategies and ensuring that at‐risk individuals receive timely assessment and support.